# Long range haplotyping of paired-homologous chromosomes by single-chromosome sequencing of a single cell

**DOI:** 10.1038/s41598-018-20069-x

**Published:** 2018-01-26

**Authors:** Deng Luo, Meng Zhang, Ting Liu, Wei Cao, Jiajie Guo, Caiping Mao, Yifan Li, Juanmei Wang, Weiren Huang, Daru Lu, Shuo Zhang, Zhoufang Li, Jiankui He

**Affiliations:** 1 Department of Biology, South University of Science and Technology of China, Shenzhen, Guangdong, 518055 China; 2 Reproductive Medicine Center, The First Affiliated Hospital of Soochow University, Suzhou, Jiangsu 215006 China; 3 Central Laboratory, Affiliated Nanshan Hospital, Guangdong Medical College, Shenzhen, Guangdong, 518052 China; 4 Department of Pediatrics, Hunan Provincial People’s Hospital, Changsha, Hunan 410005 China; 5 Key Laboratory of Medical Reprogramming Technology, Shenzhen Second People’s Hospital, The First Affiliated Hospital of Shenzhen University, Shenzhen, 518035 China; 6 State Key Laboratory of Genetic Engineering, Collaborative Innovation Center for Genetics and Development, School of Life Science, Fudan University, Shanghai, 200438 China; 7 Shanghai Ji Ai Genetics & IVF Institute, Obstetrics and Gynecology Hospital, Fudan University, Shanghai, 200011 China

## Abstract

The longest possible haplotype is chromosome haplotype that is a set of co-inherited alleles occurred on a single strand chromosome inherited from one parent. Standard whole-genome shotgun sequencing technologies are limited by the inability to independently study the haplotype of homologous chromosomes due to the short-reads sequencing strategy and disturbance of homologue chromosomes. Here, we investigated several types of chromosomal abnormalities by a dilution-based method to separate an intact copy of homologous chromosome from human metaphase cells, and then single chromosomes were independently amplified by whole-genome amplification methods, converted into barcoded sequencing libraries, and sequenced in multiplexed pools by Illumina sequencers. We analyzed single chromosome derived from single metaphase cells of one patient with balanced chromosomal translocation t(3;5)(q24;q13), one patient with (47, XXY) karyotype and one with (47, XY, 21+) Down syndrome. We determined the translocation region of chromosomes in patient with t(3;5)(q24;q13) balanced chromosomal translocation by shallow whole-genome sequencing, which is helpful to pinpoint the chromosomal break point. We showed that SCS can physically separate and independently sequence three copies of chromosome 21 of Down syndrome patient. SCS has potential applications in personal genomics, single-cell genomics, and clinical diagnosis, particularly in revealing chromosomal level of genetic diseases.

## Introduction

Human genomes are naturally diploid, and contain pairs of homologous chromosomes derived from each parent. Haplotype information is very important for understanding molecular physiology and phenotypic expression. For example, compound heterozygosity in single genes can result in different clinical conditions and disorders^1,2^. The combination of unique gene-specific haplotypes has profound effects on complex phenotypes^3^. Chromosomal balanced translocation is a disease that affects 1 in 500 humans, and is due to the rearrangement of parts of chromosome sequences between nonhomologous chromosomes^4^. Balanced translocation is difficult to be detected by regular short-read high-throughput sequencing, whereas classical karyotyping can only detect breakpoints at low resolution^5^.

Statistical and computational methods have been proposed to obtain haplotype information from conventional genotype data when the family trio datasets are available^6^. Methods for determining haplotype without parental samples have also been developed, such as long fragment read technology, dilution-based haplotyping, and deep sequencing of large-insert clones^7–10^. Determining haplotype information at the single-cell level is even more challenging. Microfluidic devices combined with SNP array genotyping were reported to obtain whole-genome phasing information^11^. Partial haplotype information can also be obtained in a single cell by chromosome microdissection methods and SNP array genotyping^12^. Single chromosome sorting developed by Yang, H., X. Chen, *et al*. can be used to analyze single chromosome, but not capable of identify the paired-homologous chromosome in single cells^10^. More recently, 10x Genomics developed the method to build human diploid de novo assemblies with phase blocks longer than 2.5 Mb by partitioning ~1ng (about 160 cells) high molecular weight DNA by 10x Genomics microfluidic platform and sequencing to 56x on HiSeq instruments^13^. However, since multiple cells were used, it lost the different characteristics from single cells and it was hard to phase across the entire chromosome because it would be hindered by the low heterozygosity region and the centromere region. The 56x sequencing depth and the specific 10x Genomics platform added up the cost too. Single-cell DNA template strand-seq to phase diploid genomes in single cells had been reported by Porubsky *et al*.^14^. It made use of the random nature of passing Watson and Crick stands from parental cells to offspring cells and introduced BrdU so as to remove the newly synthesized strands. However, waiting the single cells to separate into the daughter cells and the introducing of BrdU added the complexity of the experiment. It was difficult to reveal the chromosomal translocation events in the single cells because the whole diploid genome was in the same sequencing pool. However, most of these existing methods use multiple cells as the starting material, or they may need special devices or chips^11,15,16^, which limits the practical applications of these methods.

In this report, we applied a simple dilution-based method to perform single chromosome sequencing (SCS) of single cells. SCS physically separated and sequenced single chromosomes from a single cell. Although the conception for dilution-based technique has been reported by other groups for chromosome sequencing, there is no report published so far determining the single cell single chromosome haplotyping, which can simultaneously obtained the paired homologous chromosomes of a single cell. We demonstrated the clinical value of SCS method in identifying the chromosomal abnormal.

## Results

### SCS by dilution, amplification, and sequencing

To assess whether diluting chromosomes from a single cell to multiple pools can separate homologous chromosomes, we performed computer simulation to determine the probability of perfectly separating homologous chromosomes (Fig. S1). The simulation data indicated that diluting a metaphase-single cell in 8–24 tubes is recommended for SCS experiments to separate homologous chromosomes (Fig. S2).

**Figure 1:**
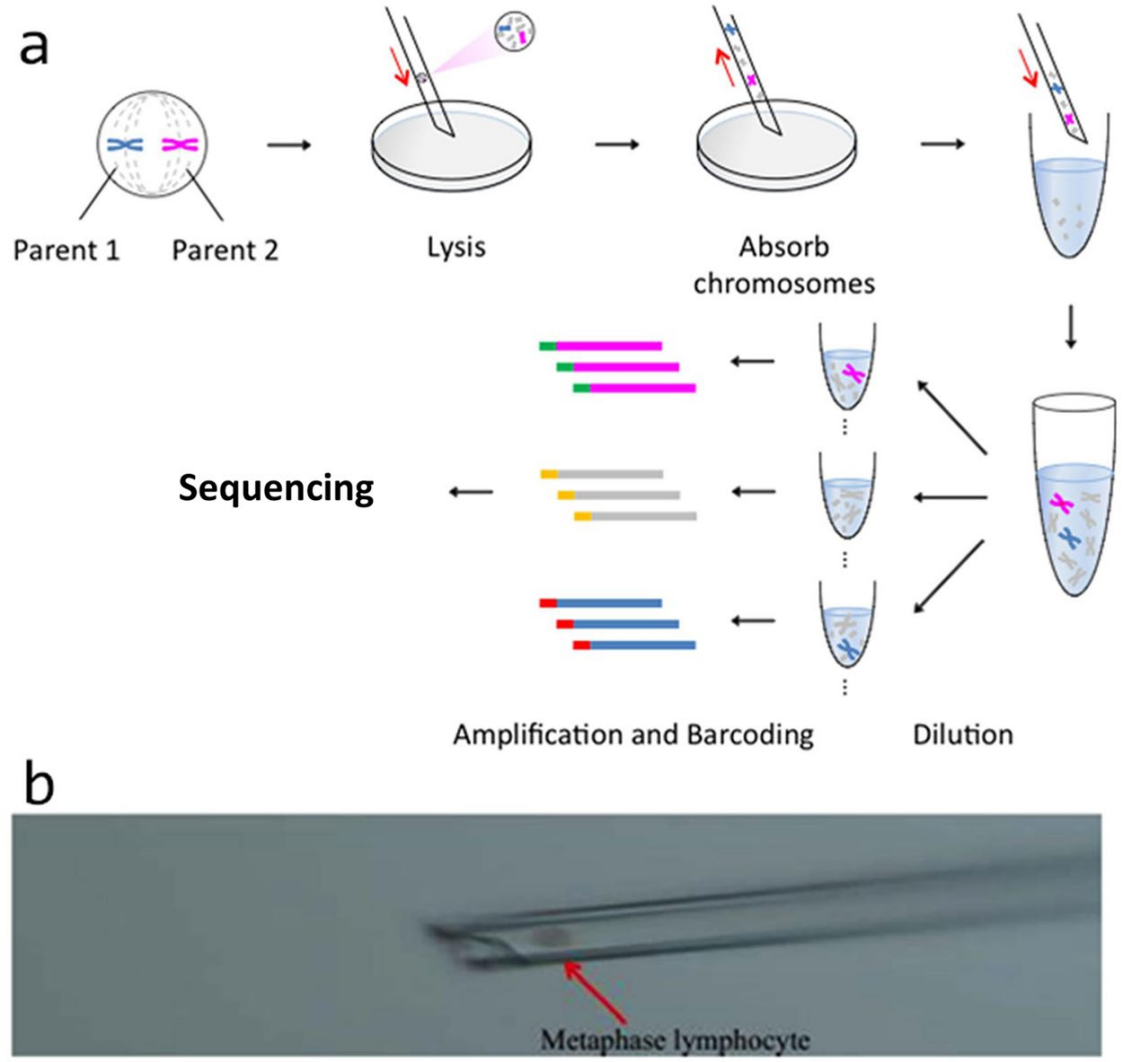
(**a**) Schematic diagram of single-chromosome sequencing work flow. First, a single metaphase lymphocyte cell that contained homologous chromosomes (one pair of homologous chromosomes is shown in blue and red) was selected by a microinjection system. Second, the cell was carefully transferred to a drop of cell lysis buffer, and the chromosomes were released from the membrane. After a few seconds, the lysis buffer was transferred to a low-binding PCR tube. Third, serial dilution was performed, and the chromosomes were separated into multiple tubes. Fourth, each tube was subjected to whole-genome amplification, next-generation sequencing library construction, index barcoding, and sequencing. (**b**) An image of a metaphase cell selected with a microinjection system.

We performed a wet lab experiment to test the method (Fig. 1). We picked individual metaphase cells by microinjection system. We lysed the single cells and performed serial dilutions into 4, 8, 16, 24, or 32 tubes respectively (Fig. S3). The mapping ratio in 8-pools (mostly > 60%) was much higher than that of the 32-pools (mostly < 30%) so as to the number of clean reads. It did seem that more chromosomes yielded more reads, like in the healthy control sample (HC) (Table 1–3, because pool6 had the highest number of reads and correspondingly the highest number of total chromosomes (Table S3). The number of mapped reads showed better correlation with the total number of present chromosomes, although it was not strictly mathematically proportional.

We observed that dilution into eight tubes was the most cost-effective and also worked best in our experiment (Fig. S4). If 23 pairs of chromosomes were arbitrarily deposited into eight tubes, 20 pairs of homologous chromosomes were expected, on average, to be physically separated into different tubes. The DNA in each tube was subjected to whole-genome amplification, followed by sequencing library construction and sequencing on Illumina sequencer. Bioinformatics pipelines were developed to identify the chromosomes in each tube (Fig. S5).

**Figure 2:**
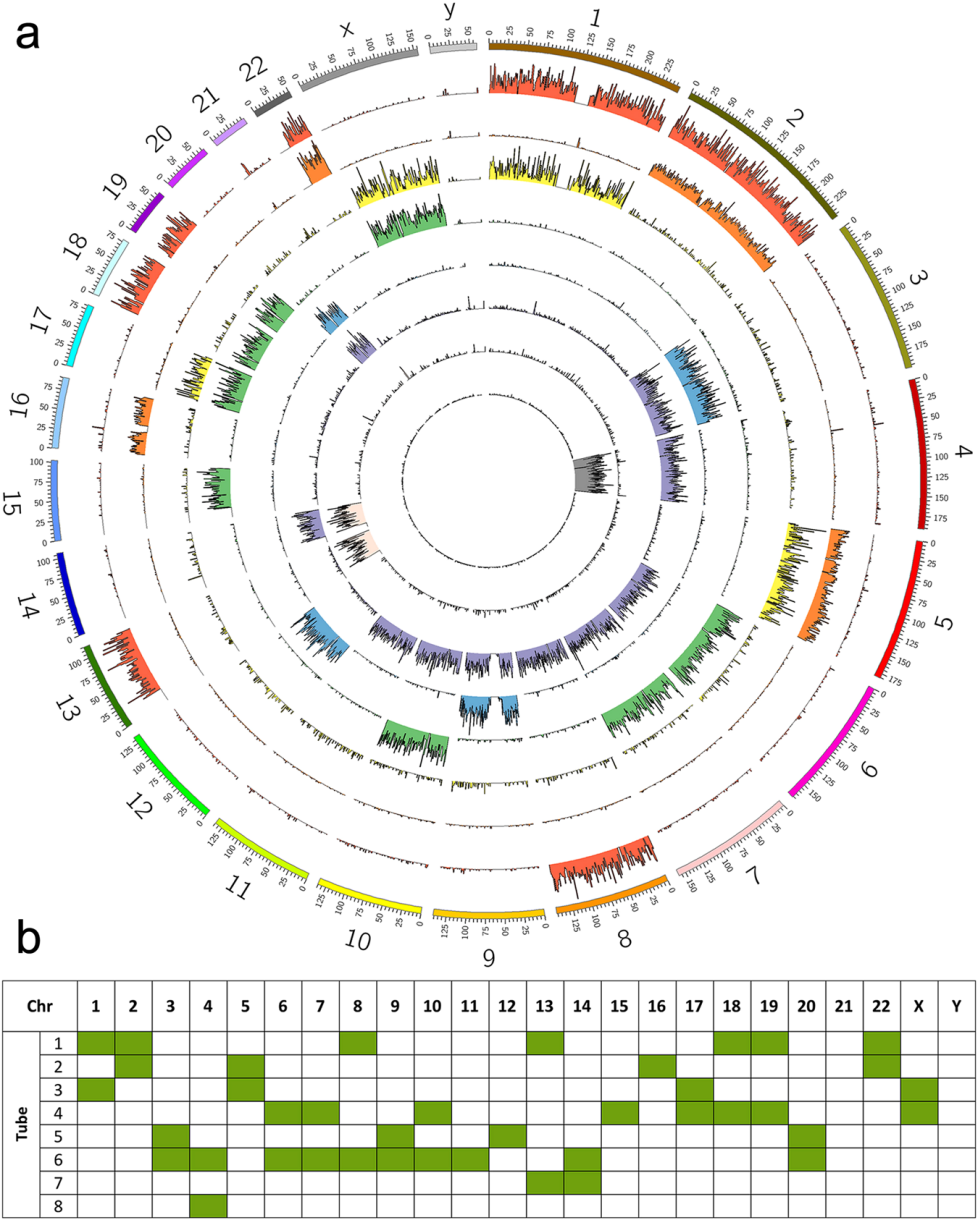
Whole-genome haplotyping. (**a**) The chromosomes from a single cell were diluted into eight tubes and sequenced. Each ring in the Circos plot represents data from one tube, and there were eight rings in total. The length of each line represents the mapped reads of each 1 million-bp window. Whether a chromosome was found in a tube was determined by the quantity of mapped reads. (**b**) Table that summarizes the chromosomes found in each tube. Eighteen pairs of homologous chromosomes were successfully separated. Chromosomes 11, 12, 15, 16, and 21, each was found in one tube or one copy was missing. The missing chromosomes may have been lost because of binding to the tube, or during transfer from the dish to the tube.

In Fig. 2, we show one example of an SCS experiment. The sample is from a white blood cell of a healthy individual. The majority of homologous chromosomes were separated into different tubes after mapping to the reference genome hg19 (Fig. 2). Forty chromosomes were recovered from the sequencing data, and six chromosomes were missing. The missing chromosomes may have been lost because of binding to the PCR tubes or tip. Eighteen pairs of chromosomes were successfully separated. Therefore, to recover all 23 pairs of chromosomes, the experiment must be repeated for two or more cells. The chromosomes were intact, because the sequenced reads were distributed throughout entire chromosomes, and no fragmental breakdown was observed (Fig. 2a). The coverage of a single chromosome varied from 5% to 22%, depending on amplification method and sequencing depth (Fig. S6).

### Identifying the chromosome breakpoint in a balanced chromosomal translocation patient T35

**Figure 3:**
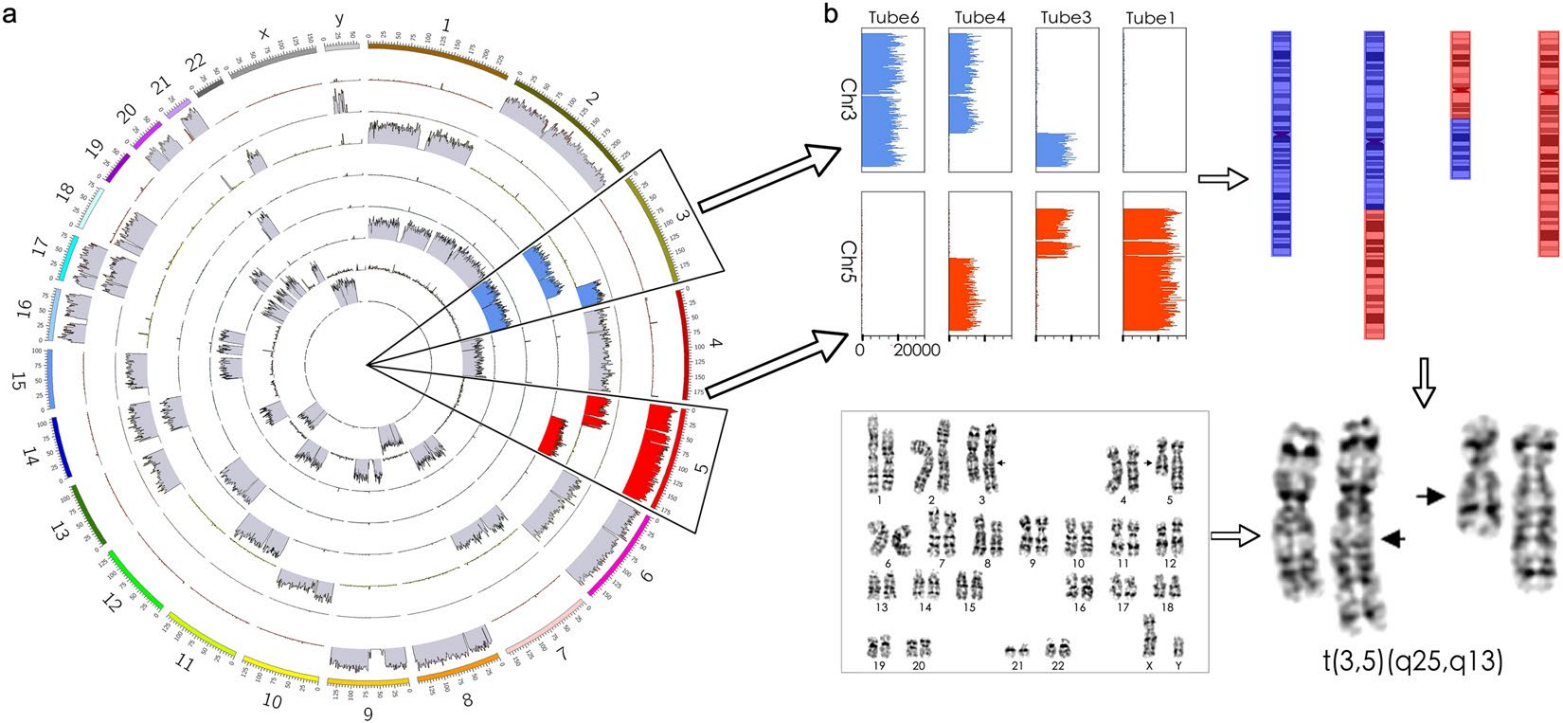
Single-chromosome sequencing (SCS) identified the balanced chromosomal translocation of a patient sample by shallow sequencing in an Illumina Miseq. (**a**) Circos plot of the SCS results of the patient sample. Each ring represents data from one tube, and eight tubes are ranked from the outer layer (tube 1) to inner layer (tube 8). Chromosomes 3 and 5 are shown in blue and red, respectively. (**b**) One copy of intact chromosome 3 was found in tube 6, whereas two fragments of partial chromosome 3 were found in tubes 3 and 4. One copy of intact chromosome 5 was found in tube 1, whereas two fragments of partial chromosome 5 were found in tubes 3 and 4. The schematic diagram of the balanced translocation of chromosomes 3 and 5 was reconstructed using SCS results. SCS identified the correct translocation, and this was validated by karyotyping results from the same patient; the SCS result indicated that the translocation was at t(3,5)(q24,q13), whereas karyotyping analysis showed that the translocation was at t(3,5)(q25,q13).

A single cell was selected and diluted into eight tubes. Shallow sequencing using an Illumina Miseq sequencer was performed to identify the translocation sites. We sequenced 9.4 million reads for this sample in total, which provided 0.26x coverage of the genome. We predicted, using SCS, that the translocation site was at t(3;5)(q24;q13) (Fig. 3 and Table S4). A repeat experiment was performed to confirm the reproducibility of SCS method (Fig. S7 and Table S3). For comparison, traditional karyotyping analysis was performed (Fig. 3b). The karyotyping analysis result, t(3;5)(q25;q13), was consistent with the SCS results, with a slight difference. SCS predicted that the translocation site was at the end of q24 but close to q25, whereas the karyotyping analysis predicted that the translocation site was at q25. SCS further narrowed down the break point to a 15-kb region in chromosome 3:148,556,760–148,571,990 and 5:73,590,780–73,605,850 (Fig. S8 and Tables 5–7. Therefore, the SCS method provided better resolution than karyotyping methods, even with shallow sequencing.

**Figure 4:**
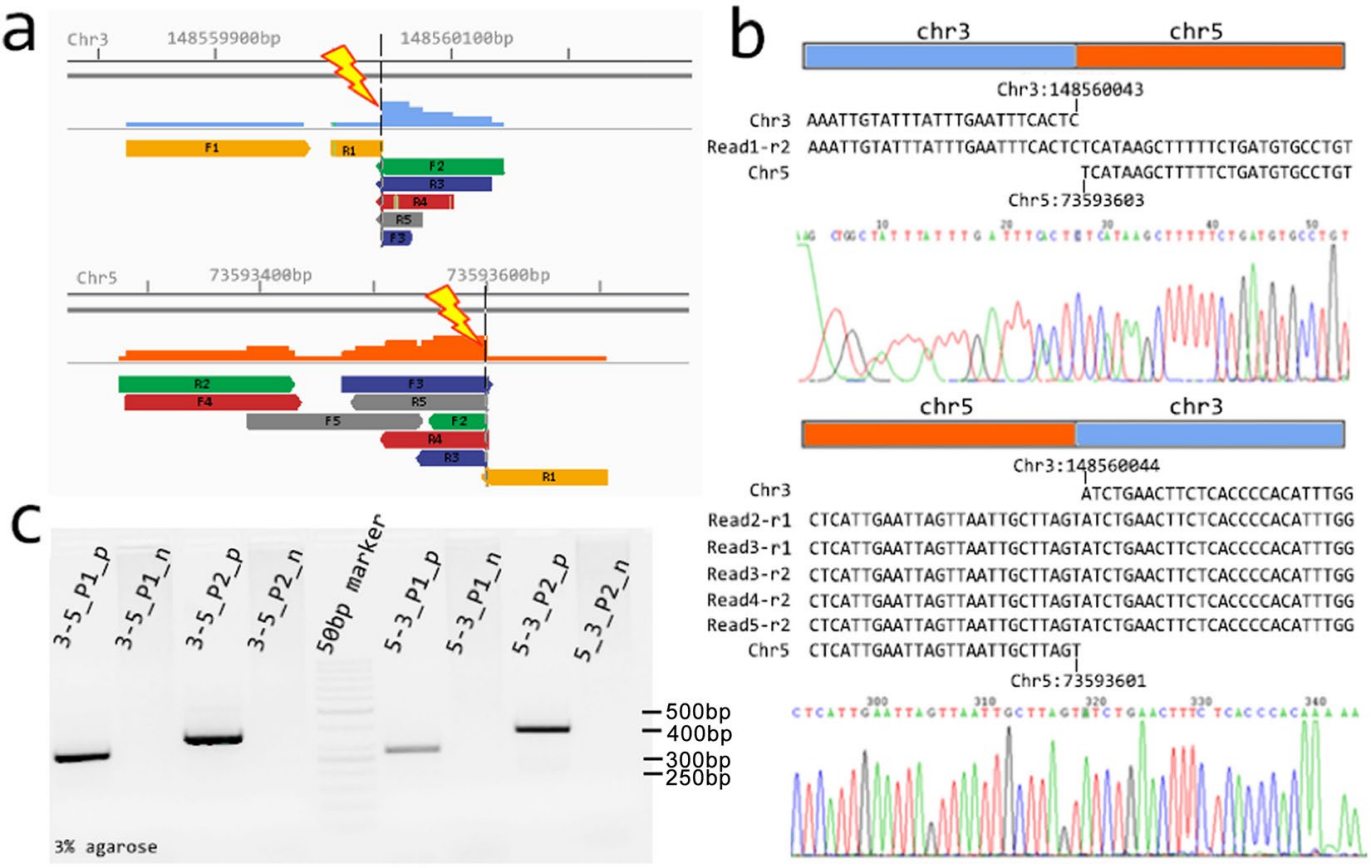
Pinpointing the chromosome break point by single-chromosome sequencing (SCS) using Illumina X10 deep sequencing. (**a**) Five chimeric reads crossed the translocation break point sequences were identified. (**b**) The patient and a normal control were further examined by PCR and Sanger sequencing. The break point is located at 148560043 for chromosome 3, and 73593601 for chromosome 5. We observed one base from chromosome 5 (73593602) that was lost during the translocation. Sanger sequencing verified the break points identified by SCS. (**c**) The break point was verified by PCR with one primer in chromosome 3 and another primer in chromosome 5, and the primers were near the break points identified by SCS. 3–5_P1_p, 3–5_P2_p, 5–3_P1_p, and 5–3_P2_p are patient samples, and 3–5_P1_n, 3–5_P2_n, 5–3_P1_n, and 5–3_P2_n are normal control samples.

We further pinpointed the break point by whole genome sequencing using Illumina X10. We sequenced 33,377,290,500 bases for this sample in total, which provided 11x coverage of the genome. We searched for chimeric reads in the chromosome 3:148,556,760–148,571,990 and 5:73,590,780–73,605,850 region that partially mapped to chromosomes 5 and 3 (Fig. 4). There were five chimeric reads that crossed the breakpoint; thereafter, the breakpoint was identified to be at position 148,560,043 in chromosome 3, and position 73,593,601 in chromosome 5 (Fig. 4a). This result was further validated by Sanger sequencing after PCR with a forward primer in chromosome 3 and a reverse primer in chromosome 5 near the break point (Fig. 4b,c). We found that one of the break points is located in the CPB1 gene region. Mutant CPB1 was previously reported to be related to infertility^17^.

### Long-range haplotype phasing

Although great progress had been made in genome phasing by both computational and experimental methods, long-range haplotype phasing is still technically challenging^18^. In SCS, because the homologous chromosomes are physically separated, we could obtain haplotype phasing information of the entire chromosome.

**Figure 5:**
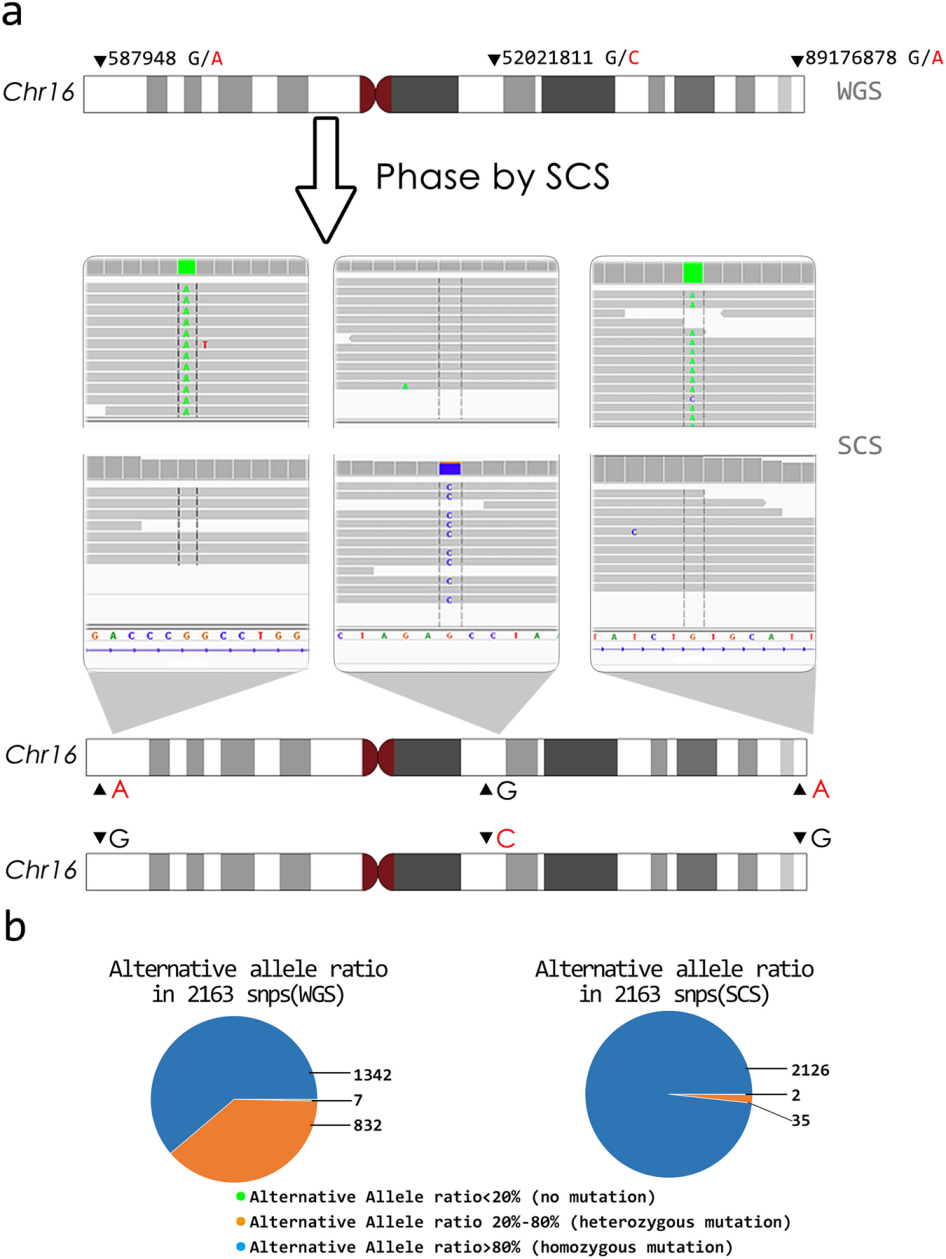
Determining the haplotype phase of loci in one chromosome by single-chromosome sequencing. (**a**) Three distant loci in chromosome 16 are demonstrated. Whole-genome sequencing of bulk cells determined the genotypes of these three loci (G/A for position 587948, G/C for position 52021811, and G/A for position 89176878). By separating the chromosomes and performing SCS, the haplotype phase of these three loci were resolved. In one copy of chromosome 16, the nucleotide at position 587948 was A, the nucleotide at position 52021811 was G, and the nucleotide at position 89176878 was A. In another copy of chromosome 16, the nucleotide at position 587948 was G, the nucleotide at position 52021811 was C, and nucleotide at of position 89176878 was G. (b) Alternative allele ratio measurement was used to decide whether a locus has a homozygous mutation (>80%), heterozygous mutation (20–80%), or is wild type (<20%). The alternative allele ratios for 2163 SNPs were calculated. Here, only the common SNPs in the 1000 Genome Project were considered. The figure on the left shows the whole-genome sequencing bulk sample results, and the figure on the right shows the SCS results. Because the SCS result was from a single haplotype, heterozygous mutations were rarely found.

We demonstrated long-range haplotype phasing using SCS by phasing three distant loci in chromosome 16 (Fig. 5). In SCS, few heterozygous mutations were observed, because only one copy of a homologous chromosome was present in each tube (Fig. 5b). SCS has relatively low genome coverage. Therefore, phasing all loci in an entire chromosome requires multiple experiments.

### Detecting chromosomal abnormality in XXY and Down syndrome patient

**Figure 6:**
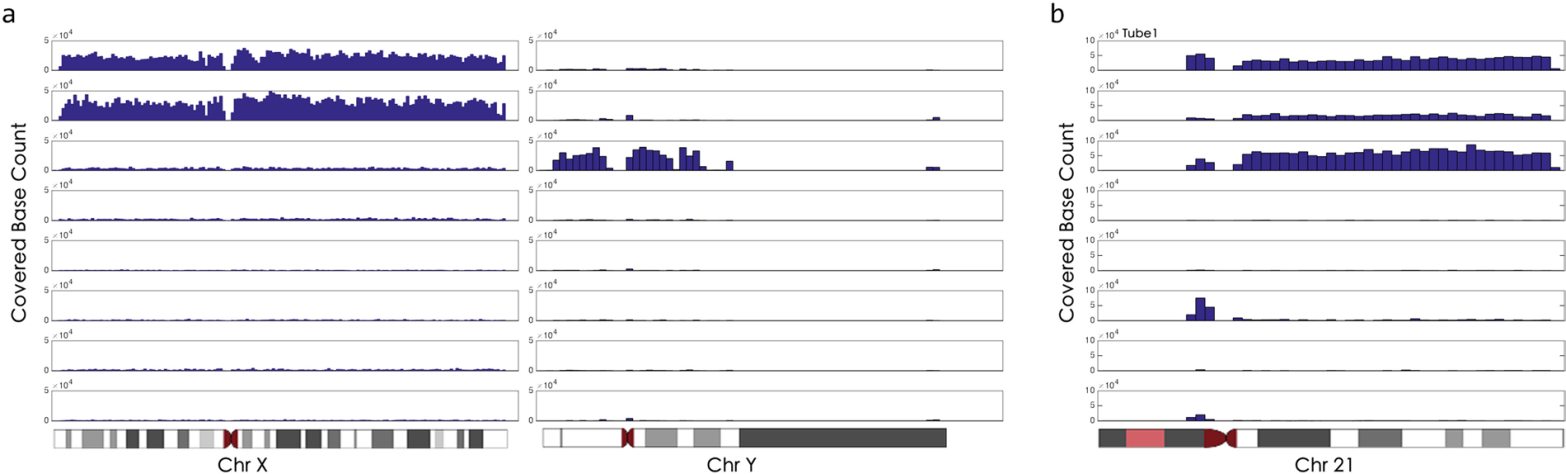
Single-chromosome sequencing (SCS) analysis of an XXY chromosome abnormal patient and a Down syndrome (T21) patient. (**A**) SCS separated two X chromosomes and one Y chromosome in the XXY patient, and (**B**) the three copies of chromosomes 21 in Down syndrome.

To demonstrate other potential use of SCS in clinical and basic research, we performed SCS on a sample with XXY chromosomal abnormality [47(+X)] and a Down syndrome sample [47(+21)] (Fig. 6 and Fig. S9). Two X chromosomes were detected in tubes 5 and 8, and one chromosome Y was detected in tube 3 for the XXY patient (Fig. 6A). For the Down syndrome sample, the three chromosome 21 each was found in tubes 1, 6, and 8 (Fig. 6B). By separating the three copies of chromosome 21, this method may help us better understand the genetics of Down syndrome.

## Discussion

We developed SCS of a single cell. This method physically separated homologous chromosomes from a single cell, amplification of the whole genome, and sequencing. SCS had less genome coverage compared with single-cell sequencing, because SCS had only one copy of each chromosome, hence with less chance to be captured, however, the accuracy is very good, similar as single-cell sequencing. SCS deep sequencing phased 5.48% of the SNPs and achieved an average accuracy of 97.72% (Table S4). Besides, we performed the analysis thoroughly which included the WGS result and a relatively deep sequencing results from four pools of a chromosomal translocation sample (T35). Among the four deep sequencing pools of SCS, eight homologous chromosomes could be phased (Table S8). The WGS data included 33,377,290,500 bp, and the equivalent sequencing depth was 11×. The coverage should be increased if we further increase the depth of sequencing. The reads were processed through the first part of the SCS analysis pipeline.

Then, for the mapped reads with mapping quality scores >30, we used GATK best practice to find germline SNPs. For WGS data, we filtered the SNPs by keeping only those with a depth >10 and quality >30. We further filtered the SNPs in WGS by retaining only those records that were found to be common SNPs (≥1% minor allele frequency in at least one 1000 Genomes population and was found in two or more people in that population) in dbSNP build149. The records after filtering served as reference heterozygous SNPs that could be phased by SCS deep sequencing. These findings served as a proof-of-concept that SCS can be used to phase the SNPs in a single cell. In principle, we could perform SCS for up to 50–100 cells from one sample and obtain higher phasing coverage. By doing so, we would lose the uniqueness of each cell, which is of critical use for research such as studies on cell lineages and cancer cell subtypes. While we find it valuable that SCS can actually separate intact physical copies of the homologous chromosomes, we admit that further improvements need to be done to increase amplification efficiency when starting from a tiny amount of initial DNA, such as reduction of the reaction volume or modification of other experimental settings and parameters.

In addition, physically separating the homologous chromosomes has advantages over other more complex labeling methods (such as including BrdU or massively barcoding multiple cells). Moreover, SCS will be more available for clinical use when all target pools are freely available (it is not possible to only select target chromosomes for further analysis with other approaches). Time and cost will also not be drastically increased because constructing eight libraries takes a similar amount of time as constructing one library, and the cost of library construction is slightly increased (approximately 25 US dollars/library); however, once we have determined which pools contain the chromosome of interest, we can dramatically reduce sequencing cost by only deep sequencing the target pools.

This method will have broad application in biological and medical research. First, by combining results with bulk-sample deep sequencing data, SCS provides whole chromosome-level haplotype phasing. Current computational methods usually require population genetic information, and current experimental methods can phase up to 90–97% of a genome^7^. In theory, SCS can phase 100% of the genome without population genetics information.

Second, SCS provides a valuable tool for studying chromosomal abnormality diseases such as Down syndrome. By physically separating the three copies of chromosome 21, it is possible to individually study the genetic and epigenetic differences of these three copies, which could help pinpoint key variations in gene expression patterns that cause Down syndrome symptoms. The same approach can also be used to analyze the chromosomes in genetically unstable cancer cells.

Third, SCS will help facilitate de novo assembly of complex genomes, such as plant genomes, which usually have multiple copies of chromosomes^19^. Conventional next-generation sequencing cannot distinguish multiple homologous chromosomes in plant cells. By combining SCS and NGS, scientists will be able to elucidate the individual chromosomes in plant and determine how they differ.

Fourth, SCS can also have medical applications. For example, human leukocyte antigen (HLA) haplotypes are associated with autoimmune disease and clinical outcomes after transplantation^20^. HLA has very complicated genomic regions and varies among populations, which is still a big challenge to reveal the mechanism. SCS may help provide high-resolution HLA genotyping; hence it is possible to better understand the biological mechanism of HLA-associated diseases.

SCS still requires a lot of improvement before it can be widely used. We need to further optimize the wet lab techniques to avoid chromosome loss and increase coverage (Fig. S10). Because the DNA template of single chromosome is much less than that of a single cell, the whole-genome amplification method should be optimized to improve coverage. Different single-cell amplification methods were tested to increase coverage, including the MDA, MALBAC, and Genomeplex WGA4 (Fig. S11).

## Materials and Methods

### Ethics

This study was approved by the Southern University of Science and technology of China (SUSTC). All the experiments were performed in accordance with guidelines and regulations of the SUSTC. All methods are approved by the committee of SUSTC and carried out in accordance with relevant guidelines and regulations. All the analysis was performed anonymously. Written informed consent was obtained from each participant.

### Patients and Blood samples

Human blood sample of Healthy Control sample (HC) was obtained from Nanshan hospital, Shenzhen. Human blood sample of Down syndrome patients [47(+21)] were obtained from Hunan Provincial People’s Hospital. Human blood sample of balanced chromosomal translocation [T35] and XXY genotyping [47(+X)] were from the First Affiliated Hospital of Soochow University Hospital. The sample information is summarized in supplementary material Table S1.

### Cell culture

Twenty-five drops of blood were added to 5 mL cell culture medium. After 69 h incubation (37 °C and 5% CO_2_), 50 μL colchicine at 40 mg/mL was added to the medium, and incubated for another 3 h.

### Metaphase lymphocyte collection

The cell culture medium was centrifuged at 500 × *g* for 5 min. The supernatant was discarded; then, 10 mL 75 mM KCl solution was added. The cells were gently suspended and kept at room temperature for 15 min. Subsequently, 200 μL acetic acid (final concentration 2%) was added and the solution was gently mixed. The cells were placed on ice for 30 min, and were centrifuged at 800 × *g* for 5 min, and the supernatant was then discarded. A 10-mL solution of ethanol and acetic acid at a 3:1 ratio was added to the cells. The cells were centrifuged at 800 × *g* for 5 min after suspension. The supernatant was then discarded. The cells were washed using 2 mL 75 mM KCl solution and centrifuged at 800 × *g* for 5 min. Cell resuspension solution (1 mM EDTA, 1% Triton-X100, 0.2 mg/mL RNase A, 75 mM KCL) were added to the cells. The cells were placed at 4 °C overnight.

### Single cells selection and lysis

Single metaphase lymphocyte cells were selected under a Zeiss Axiovert 20 microscope, with an Eppendorf PatchMan microinjection system (Eppendorf, Hamburg, Germany). A single cell was lysed in buffer (0.03% pepsin, 1% Triton-X100, 2% acetic acid, 75 mM KCL) and transferred to a low-binding PCR tube (Axygen, MA, USA) with 9 μL nucleic free water and centrifuged.

### Dilution

It is important that the dilution into multiple tubes (pools) should follow the order described in Fig. S3; we attempted different approaches to dilute the single chromosomes from individual metaphase cell. During dilution, we always use the same tip for the whole process without changing tips to avoid loss of chromosomes due to binding to the tips.

### Whole-genome amplification and sequencing library construction

We modified the protocol of the GenomePlex WGA4 kit (Sigma-Aldrich, CA, USA) to perform amplification. The detailed protocol was provided in the supplementary materials. This protocol was specially optimized for dilution in eight tubes. Dilution in more tubes may require protocol adjustment. We constructed the sequencing library for an Illumina Miseq or Hiseq X10 sequencer (Illumina, CA, USA).

### Bioinformatics analysis

We used mapping quality score as a metric to filter out low-quality reads and performed the analysis (Table S2 and Fig. S12). The sequence reads were mapped onto reference genome hg19^21^ with bwa (version 0.7.16)^22,23^. We used mapping quality score as a metric to filter out low-quality reads. To present the overall quality distribution of sequencing reads, we plotted the MAPQ distribution in Fig. S12 (MAPQ: MAPping Quality = −10 log10(P), Where P = probability that this mapping is NOT the correct one). We found that most of the reads had a MAPQ value of 60 (<10^−6^ probability that a read was incorrectly mapped), so we increased the MAPQ cutoff from 5 to 30 (<10^−3^ probability that a read was incorrectly mapped) and analyzed all data again. The resulting circos plots were very similar to the previous plots, and we could still distinguish individual chromosomes. For per base quality, we used trimmomatic to clean our raw data with the option “SLIDINGWINDOW:4:15,” which means that, for every four consecutive bases, the average base-calling quality (phred score) must be >15. By employing this parameter, we observed that all bases’ phred scores were >30 (less than 10−3 probability that a base was incorrectly called), as depicted in Fig. S13, which gave us high confidence in the downstream analysis.

The mpileup file was generated by Samtools (version 1.3.1)^24^, the mapped bases in each 1 million-bp window were counted, and a Circos (version 0.67–7)^25^ plot was generated to show the distribution of reads across all chromosomes for all tubes. The GC content bias and genome coverage uniformity were also investigated. To call variants, BWA (version 0.7.16)^22^ and GATK (version 3.8.0)^26^ best practice were used.

### Availability of data

Data are available from NCBI BioProject database under accession number [PRJNA419806].

## Electronic supplementary material

Supplementary Materials
