# Supplementary material for: Long range haplotyping of paired-homologous chromosomes by single-chromosome sequencing of a single cell
Source: Sci Rep. 2018 Jan 26;8:1640. doi: 10.1038/s41598-018-20069-x (PMC5785984; doi:10.1038/s41598-018-20069-x)
Supplement: Supplementary file 1 — Supplementary Materials [file 41598_2018_20069_MOESM1_ESM.pdf]

Supplementary Materials

Long range haplotyping of paired-homologous chromosomes by single-chromosome sequencing of a single cell

Deng Luo[1#], Meng Zhang[1#], Ting Liu[1], Wei Cao[1], Jiajie Guo[1], Caiping Mao[2], Yifan Li[3], Juanmei Wang[4], Weiren Huang[5], Daru Lu[6], Shuo Zhang[7], Zhoufang Li[1]* and Jiankui He[1]*

[1]Department of Biology, South University of Science and Technology of China, Shenzhen, Guangdong, 518055, China

[2]Reproductive Medicine Center, The First Affiliated Hospital of Soochow University, Suzhou, Jiangsu, 215006, China

[3]Central Laboratory, Affiliated Nanshan Hospital, Guangdong Medical College, Shenzhen, Guangdong, 518052, China

[4]Department of Pediatrics, Hunan Provincial People's Hospital, Changsha, Hunan, 410005, China

[5]Key Laboratory of Medical Reprogramming Technology, Shenzhen Second People's Hospital, The First Affiliated Hospital of Shenzhen University, Shenzhen, 518035, China

[6]State Key Laboratory of Genetic Engineering, Collaborative Innovation Center for Genetics and Development, School of Life Science, Fudan University, Shanghai, 200438, China

[7]Shanghai Ji Ai Genetics & IVF Institute, Obstetrics and Gynecology Hospital, Fudan University, Shanghai, 200011, China

[#] Equal contribution

*Corresponding author: hejk@sustc.edu.cn; lizf@sustc.edu.cn

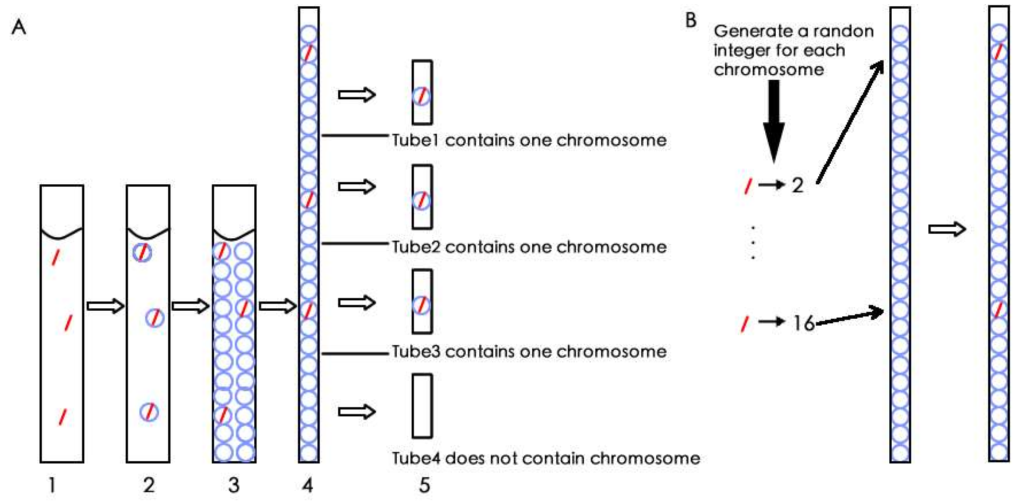

Figure S1 The computer simulation process

A simulation was performed to assess how many homologous copies of chromosome will be separated by dilution. Here is the algorithm for the simulation:

1. Assume chromosomes are balls with the same volume. Assume water is also divided into multiple balls with the same volume as the chromosome ball. Let assume there are “m” balls in total. Therefore, in a tube, there are two kinds of balls, one with chromosome, the others without chromosome.

2. The dilution process is simulated by distributing balls to different tubes.

3. We randomly choose 46 balls to mark the chromosome number on them. The random number is generated by *rand()* in perl

4. We distribute the balls into “n” different tubes sequentially, here n=4, 8, 16, 24, 36….

5. The number and types of the chromosome in each tube is counted. During each simulation experiment, we calculated: (1) how many homologous chromosome pairs failed to be separated, and appeared in the same tube; (2) how many experiment successfully separated all the homologous chromosomes; (3) how many tubes contained only one chromosome; 4) how many tubes didn’t contain any chromosome.

6. The simulation was performed 100 times for each parameter setting to calculate the average value.

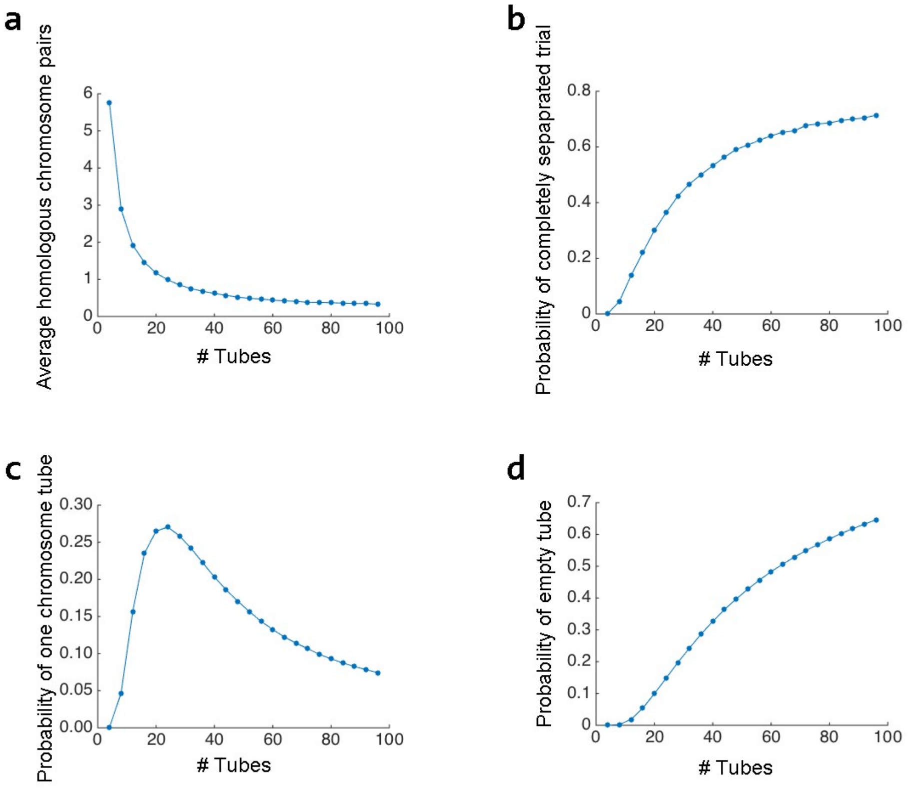

Figure S2 Simulation results. (a) Average homologous chromosome pairs that are not separated successfully were shown. When we dilute to 8 tubes, there will be 2.89 homologous chromosome pairs that are not separated successfully; (b)The probability of the experiments that separate the homologous chromosomes completely; (c)probability of the tubes that contain only one homologue chromosome; (d) probability of the tubes that do not contain any chromosome.

As shown in Figure S2, the more tubes used in dilution, the better results of separating homologous chromosomes would get. However, the more tubes used in the experiment, the higher cost for SCS sequencing as well as more challenges for amplification because few DNAs templates were in each tube. Therefore, we recommended 8-24 tubes dilutions.

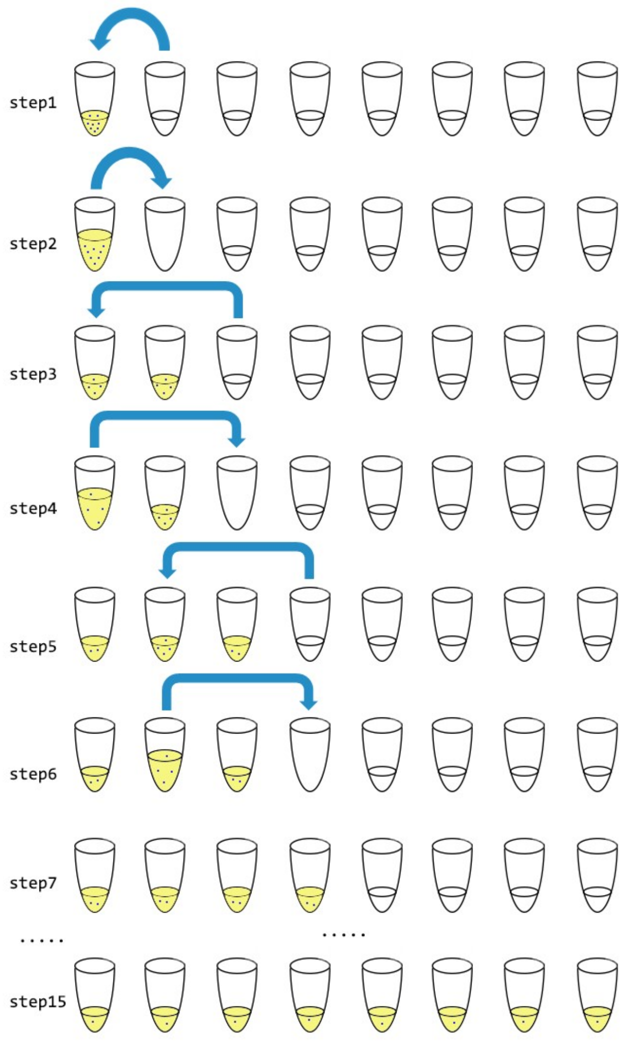

Figure S3 Dilution procedure. We lysed a single cell in the microcapillary and immediately transferred to one low-binding PCR tube. Then the dilution procedure was followed. To minimize the chance of DNA binding to the wall of PCR tubes, and to increase the uniformity of chromosome distribution among tubes, we optimized the dilution procedure. We tried different dilution procedures; however, the procedure presented here got the best results.

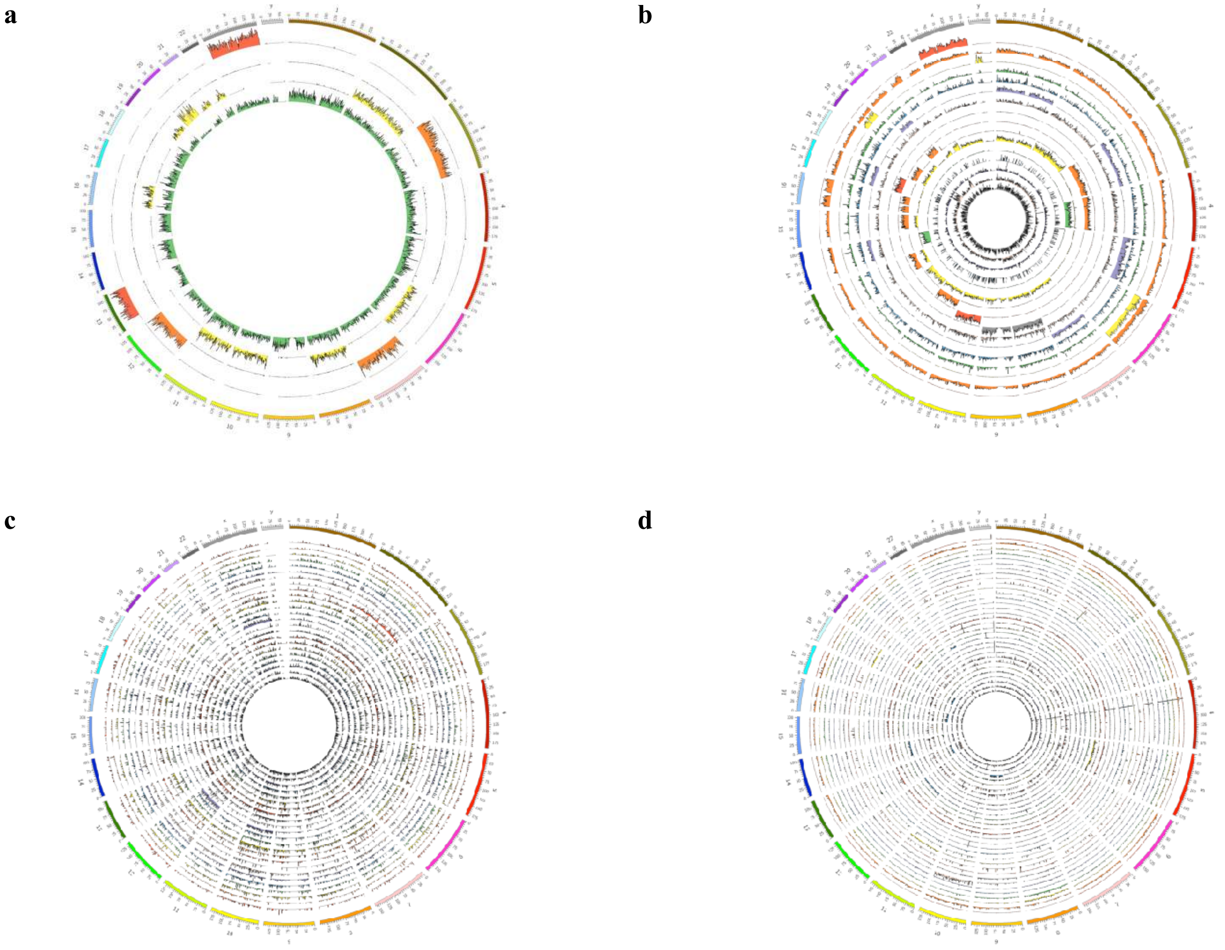

Figure S4. Circos plot for diluting into 4 tubes (a), 16 tubes (b), 24 tubes (c) and 32 tubes (d).

Dilution in 4, 16, 24 and 32 tubes

We tried to dilute a single cell into 4, 16, 24 and 32 tubes. According to our simulation, the more tubes used in dilution, the less chance that homologous chromosome pairs will appear in the same tube. In Fig. S4, the Circos plots for 4, 16, 24, and 32 tube dilution results were shown. When 32 tubes used in dilution, we found that the majority of tubes contained no chromosome or failed in genome amplification (Fig. S4d). This can be explained that less DNA leads to lower amplification efficiency. Another reason is that more dilutions lead to chromosomes fragmentations. We chose 8-tube dilution strategy in this paper, because it is relatively easy and cost-effective, and has a high success rate in genome amplification.

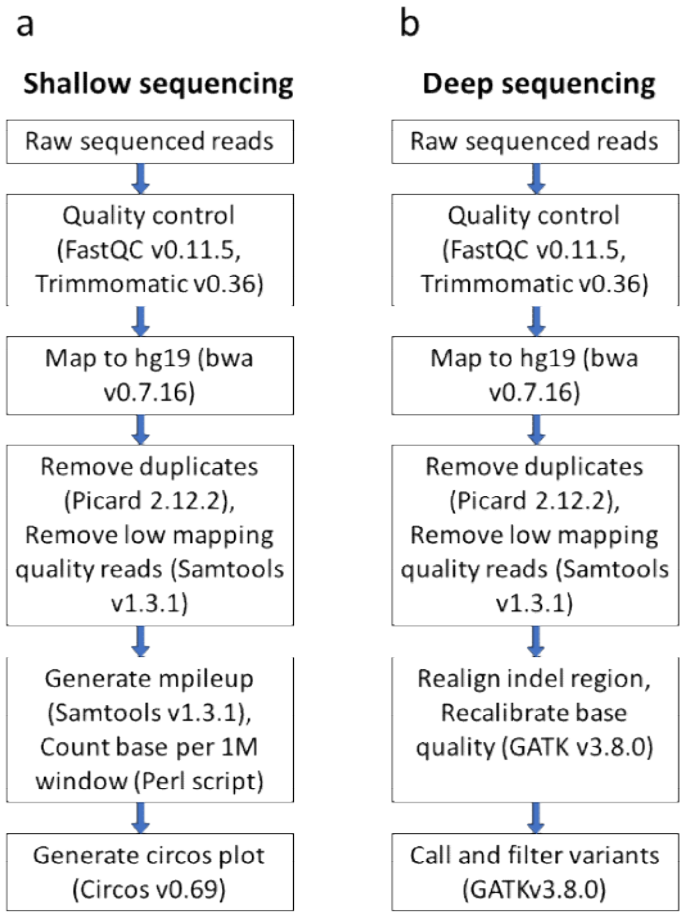

Figure S5. Bioinformatics pipeline (a) The bioinformatics workflow and software used in shallow sequencing by Illumina Miseq. (b) The bioinformatics workflow and software used in deep sequencing by Illumina Hiseq.

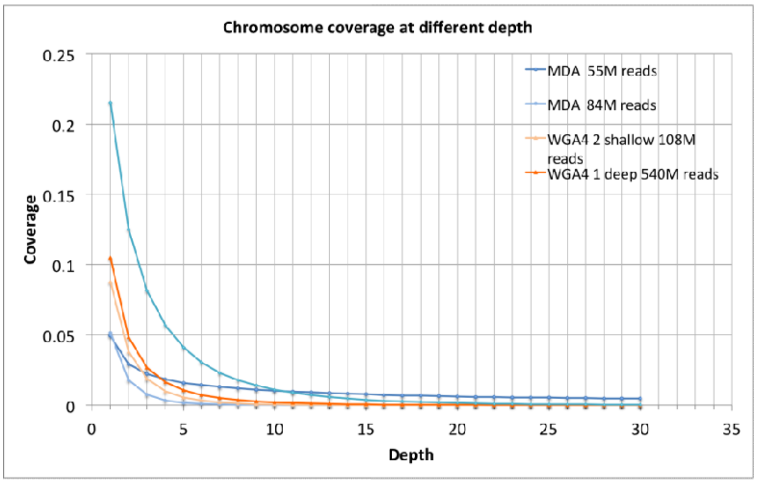

Figure S6. Chromosome coverage of different amplification methods and at different depth. We compared the genome coverage of single chromosome sequencing using two different whole genome amplification methods: GenomePlex WGA4 and MDA. As we only sequence them with very low depth, most of regions covered only one reads.

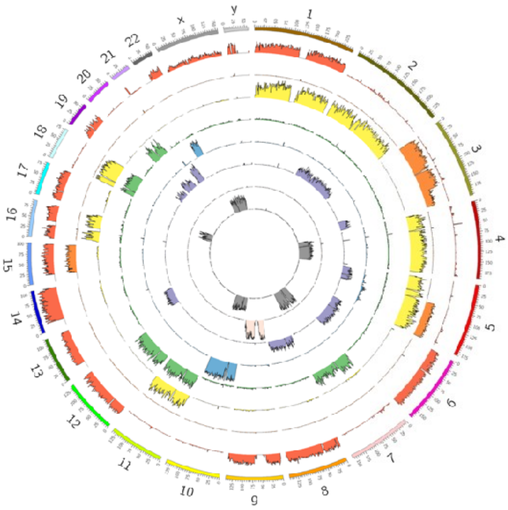

Figure S7. Circos plot of T35-repeat.

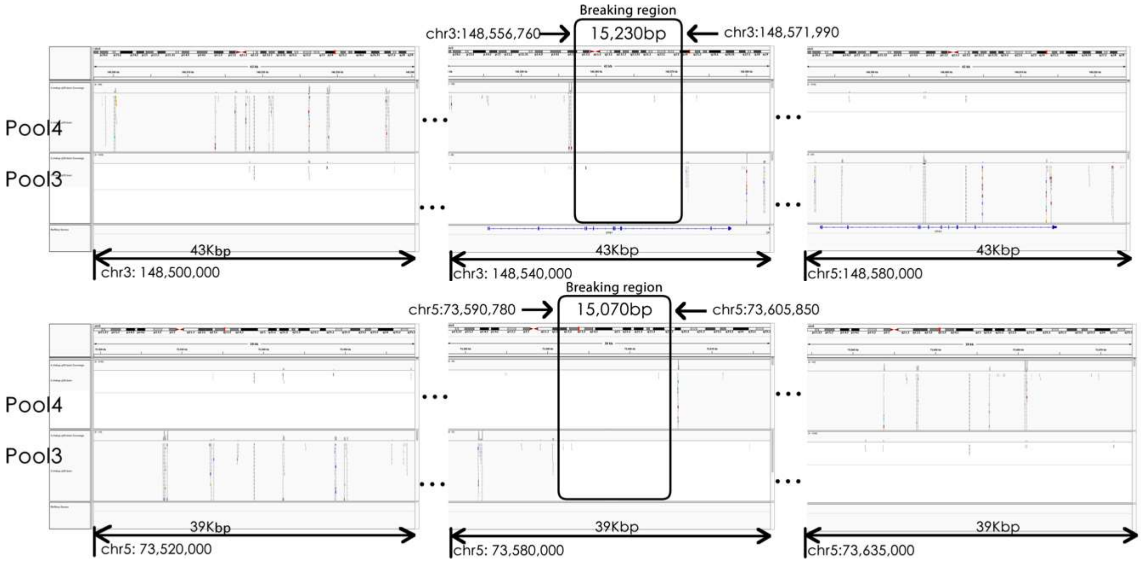

Figure S8. Deep sequencing from SCS to narrow down break points in 15Kb window

a **b**

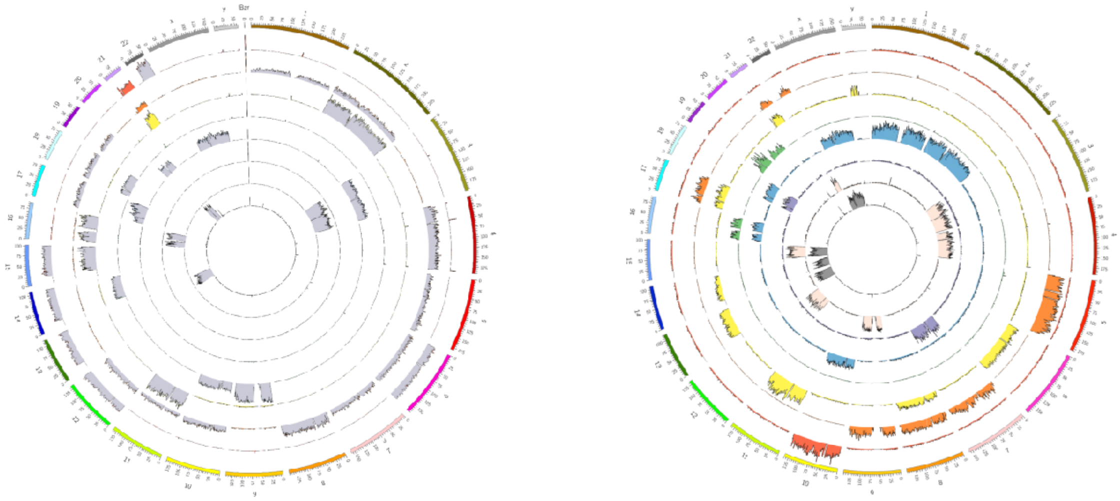

Figure S9. Circos plot for Down syndrome sample (a) and XXY sample (b)

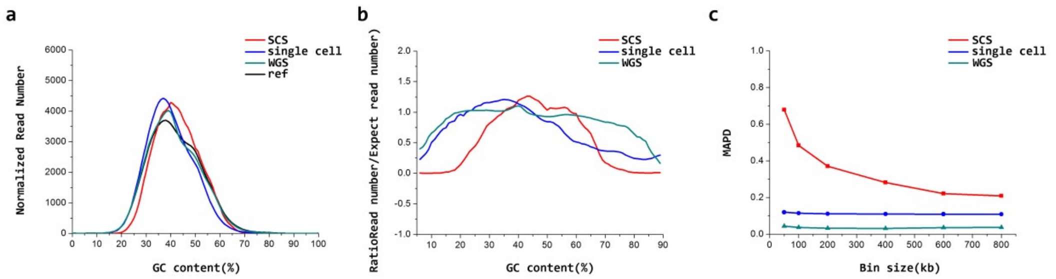

Figure S10. (a) The GC content histogram of four methods (single chromosome sequencing sample, single cell sample, bulk-cell sample and simulation by hg19 reference). The y-axis was normalized reads with different GC contents. The number of total reads of each sample was normalized to 100000. (b) The GC-bias plot. The relative coverage (y-axis) represented the ratio of the coverage of a sample to the coverage predicted by the reference genome. A relative coverage of 1 indicated no bias. A relative coverage above 1 or below 1 indicated higher or lower coverage than that expected. (c) MAPD metric to determine the detection limit of CNVs. The MAPD is calculated in various bin size from 50 kb to 800 kb.

GC bias

We calibrated the GC content bias of SCS by comparing to that of single cell sequencing, bulk cell whole genome sequencing, and simulated reference data. The GC bias of SCS was strongly affected by the whole genome amplification methods. In Figure S10, we observed that the SCS tended to have higher reads with the GC content ranging from 20%-70%. We also calibrated of the coverage uniformity of genome by the MAPD methods, which is adopted in microarray analysis. The MAPD of SCS is higher than that of single cell and bulk cell results, indicating less uniform coverage (Figure S10c).

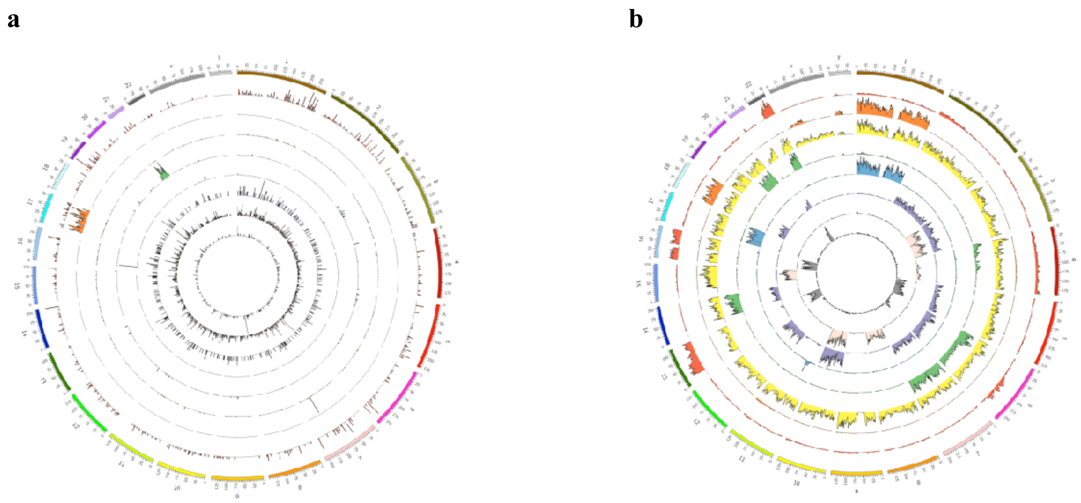

Figure S11. Circos plot for SCS data with whole genome amplification methods MDA (a) MALBAC (b)

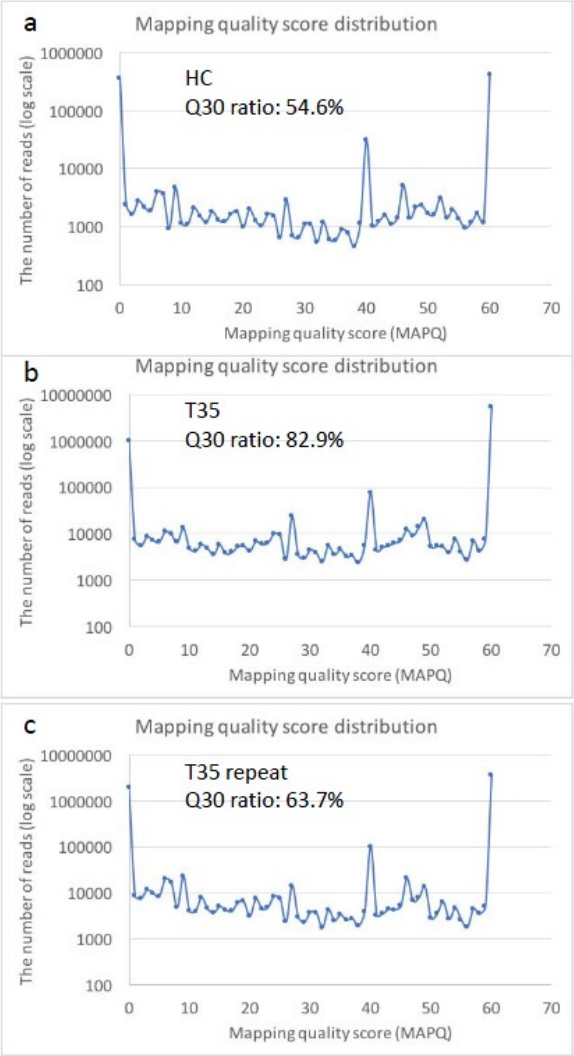

Figure S12. Mapping quality distributions for HC, T35, and T35 repeat. Mapping quality scores >30 accounted for 54.6%, 82.9%, and 63.7%, respectively, in three samples. HC was a healthy control sample whose SCS circos plot is shown in Fig. 2; T35 was a sample with a balanced chromosomal translocation whose SCS circos plot is shown in Fig. 3. Sample information is provided in Table S1.

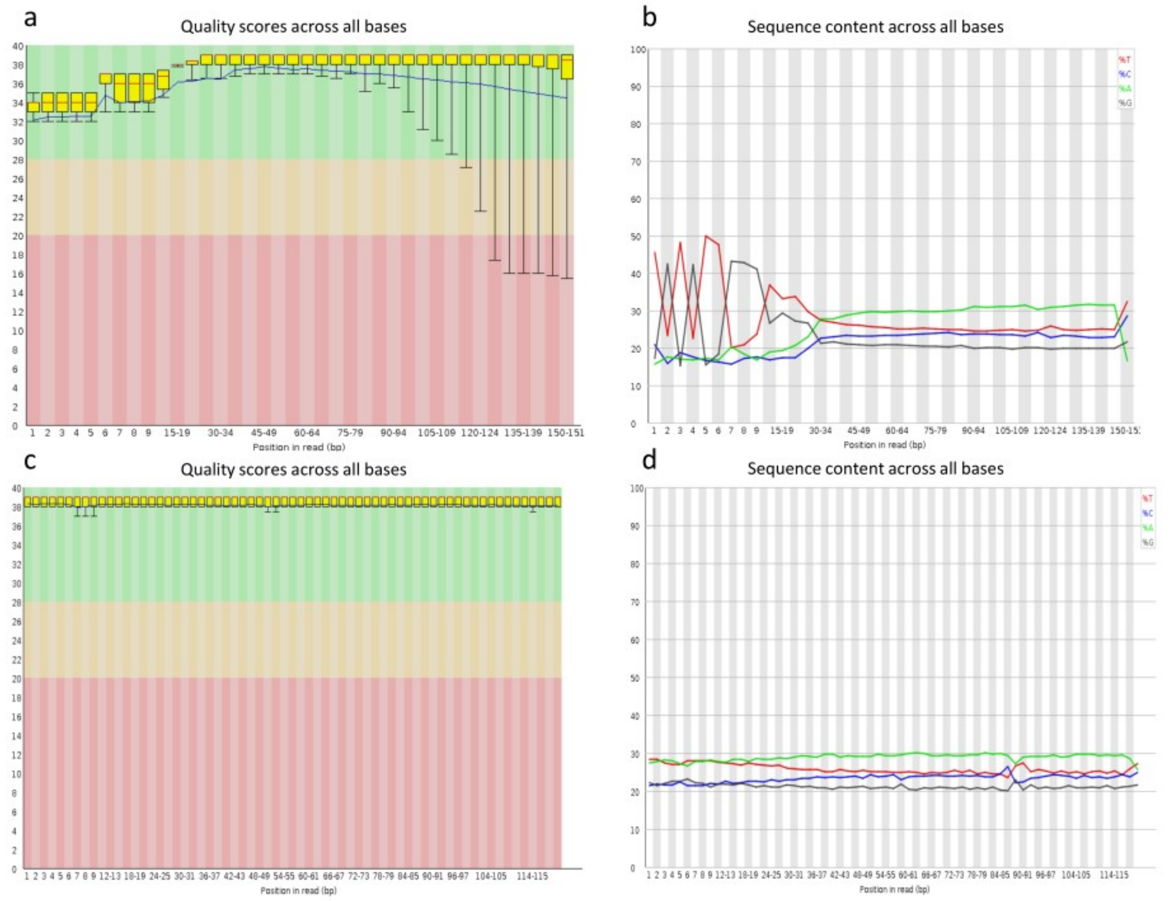

Figure S13. Per base sequence results. a) Per base sequence quality of raw sequencing reads. b) Per base sequence content of raw sequencing reads. We removed the first 30 bp and set the maximum length to 120 bp. c) Per base sequence quality of reads after quality control. d) Per base sequence content of reads after quality control.

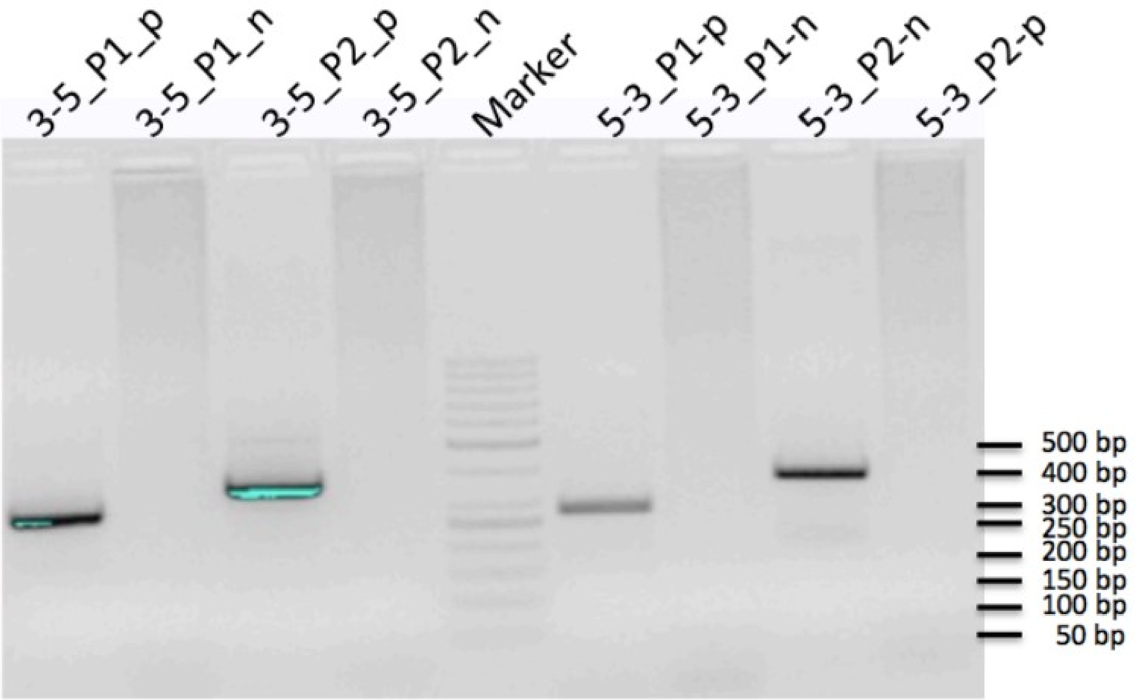

Figure S14. Original gel photo for breaking point detection

Table S1. Sample information				
**ID**	**Age (year)**	**Gender**	**Karyotyping result**	Hospital
HC	28	M	46, XY	Nanshan Hospital
T35	26	M	46,XY, t(3,5), (q25,q13)	the First Affiliated Hospital of Soochow University Hospital.
47(+X)	27	M	47, XXY	the First Affiliated Hospital of Soochow University Hospital.
47(+21)	3	F	47, trisomy 21	Hunan Provincial People's Hospital

Note:

[ID: HC]

HC is a sample from a 28-year old male

[ID: T35]

Item：human karyotype by G-Band analysis (400 bands)

Standards: ISCN 2013: An International System for Human Cytogenetic Nomenclature (ISCN 2013)

Result: 46, XY, t(3;5), (q25;q13)

[ID: 47(+X)]

Item：human karyotype by G-Band analysis (400 bands)

Standards: ISCN 2013: An International System for Human Cytogenetic Nomenclature (ISCN 2013)

Result: 47, XXY

[ID: 47(+21)]

Item：human karyotype by G-Band analysis (400 bands)

Standards: ISCN 2013: An International System for Human Cytogenetic Nomenclature (ISCN 2013)

Result: 47, trisomy 21

**Table S2. Quality control on raw sequencing data metrics**			
Sample	Raw data size (bp)	Clean data size (bp)	Equivalent sequencing depth*
HC	147,362,241	82,110,463	0.027
T35	1,650,279,649	1,188,159,349	0.39
T35-repeat	979,814,057	724,047,003	0.24
* Equivalent sequencing depth are calculated by using clean reads divide the hg19 genome total length, 3,036,303,846 (chromosome 1 to 22 and X).			

Table S3. Reads statistics across processing pipeline

**Sample**	**Pool**	**Clean reads**	**Mapped reads**	**Mapping ratio**	**Rmdup reads***	**Rmdup retain ratio**	**Total No. of Chromosomes identified**
**HC**	Pool1	153958	122693	0.796925	115380	0.940396	7
	Pool2	194044	105023	0.541233	101459	0.966065	4
	Pool3	56071	20532	0.366179	19985	0.973359	4
	Pool4	60326	40056	0.663992	39670	0.990363	8
	Pool5	76099	51617	0.678287	50308	0.97464	4
	Pool6	255893	211490	0.826478	199761	0.944541	10
	Pool7	28785	13530	0.470036	13305	0.98337	2
	Pool8	96268	78558	0.816034	76427	0.972874	1
**T35**	Pool1	1385701	1286610	0.92849	946235	0.735448	9
	Pool2	1032377	981979	0.951183	586142	0.596899	3
	Pool3	1354922	1332222	0.983246	900100	0.675638	9
	Pool4	1318922	1291307	0.979062	726415	0.562542	4
	Pool5	1242860	922656	0.742365	583095	0.631974	2
	Pool6	1478140	1439649	0.97396	947953	0.658461	6
	Pool7	1474457	1437868	0.975185	835012	0.580729	9
	Pool8	1106340	1068250	0.965571	659463	0.61733	3
**T35 repeat**	Pool1	845101	606262	0.717384	589181	0.971826	15
	Pool2	690525	551951	0.799321	456190	0.826505	3
	Pool3	667009	582573	0.873411	569811	0.978094	7
	Pool4	778636	549955	0.706306	471064	0.85655	5
	Pool5	699190	514088	0.735262	474565	0.92312	2
	Pool6	735957	537604	0.730483	497048	0.924562	8
	Pool7	792500	524308	0.661587	459287	0.875987	1
	Pool8	1073964	779733	0.726033	739775	0.948754	5

**Reads after duplicates were removed.*

Note: Circos plot for HC was shown in Figure 2, Circos plot for T35 was shown in Figure 3

Search for break point in chromosomal translocation sample T35

From shallow sequencing result of T35 (Figure 3), we first checked the covered bases in 1Mb window, and was able to narrow down the break point to chr3: 148M-149M, chr5: 73M-74M. When looking at 100Kb window, we could narrow down the break point to 200Kb window, chr3: 148400K-148600K, chr5: 73500K-73700K. With deep sequencing for pool3 and pool4 library in Figure 3, seeing from IGV, we can further narrow down the break point to chr3: 148,556,760-148,571,990 and chr5: 73,590,780-73,605,850, shown in Figure S9. Then we performed the whole genome sequencing for this sample, generated 33,377,290,500 bases, which provided 11x coverage of the genome. We searched chimeric reads that can partially map to chromosomes 3 and 5. We mapped the first 30bp from both Read1 and Read2 to the whole genome and tried to find the pairs whose Read1 and Read 2 were crossing chromosomes 3 and 5. If we do not define a targeted region, we found there were too many of potential chimeric reads that we could not find any clue of the break points, Table S7. However, when we searched only in chr3: 148,556,760-148,571,990 and chr5: 73,590,780-73,605,850, we then found 4 chimeric reads pairs. By elongating the cropped 30bp reads to their full length along the reference sequence, we found two break points on two chromosomes. We further examined the singleton reads near the break points in IGV and found one more read pair supporting those two break points. All five read pairs are shown in Figure 4.

Table S4. SNP phasing statistics for sample T35

Chr	Heterozygous SNPs found in WGS*	Phasing positions covered in haploid 1**	Phasing positions covered in haploid 2	Concordant SNPs in haploid 1	Concordant SNPs in haploid 2	Conflict SNPs	Phased SNPs	Phased Ratio	Accuracy
1	48881	1125	1216	1124	1211	31	2273	4.65%	97.10%
2	44848	1380	1290	1380	1285	16	2633	5.87%	98.61%
3	35879	690	999	690	999	29	1631	4.55%	96.57%
5	32432	1482	727	1480	727	32	2143	6.61%	97.01%
6	36971	1445	993	1441	988	17	2395	6.48%	98.24%
14	20202	444	382	443	381	0	824	4.08%	99.76%
15	18363	447	504	445	504	21	907	4.94%	95.37%
16	23867	900	707	899	705	6	1592	6.67%	99.07%
						Sum/Average	14398	5.48%	97.72%

*Heterozygous SNPs found in WGS, and filtered by common SNP in dbSNP149

**Phasing positions were the positions of Heterozygous SNPs found in WGS.

Table S5. Shallow sequencing from SCS to narrow down break points in 1-Mb window

Pool	Chr	Start	Stop	Covered bases	Breaking indicator
3	5	71000000	71999999	21508	
	5	72000000	72999999	22418	
	5	73000000	73999999	15236	*
	5	74000000	74999999	406	
	5	75000000	75999999	372	
3	3	146000000	146999999	1026	
	3	147000000	147999999	129	
	3	148000000	148999999	7978	$
	3	149000000	149999999	28885	
	3	150000000	150999999	20252	
4	3	146000000	146999999	16328	
	3	147000000	147999999	15522	
	3	148000000	148999999	11618	$
	3	149000000	149999999	667	
	3	150000000	150999999	518	
4	5	71000000	71999999	0	
	5	72000000	72999999	0	
	5	73000000	73999999	4943	*
	5	74000000	74999999	17161	
	5	75000000	75999999	14505	

Table S6. Break points located by shallow sequencing with SCS in a 200-Kb window

Pool	Chr	Start	Stop	Covered bases	Breaking indicator
3	5	73300000	73399999	1741	
	5	73400000	73499999	2044	
	5	73500000	73599999	2706	*
	5	73600000	73699999	0	
	5	73700000	73799999	0	
	5	73800000	73899999	178	
3	3	148200000	148299999	193	
	3	148300000	148399999	0	
	3	148400000	148499999	0	$
	3	148500000	148599999	944	
	3	148600000	148699999	2094	
	3	148700000	148799999	694	
5	3	148300000	148399999	1771	
	3	148400000	148499999	1111	
	3	148500000	148599999	2366	$
	3	148600000	148699999	0	
	3	148700000	148799999	0	
	3	148800000	148899999	0	
5	5	73300000	73399999	0	
	5	73400000	73499999	0	
	5	73500000	73599999	0	*
	5	73600000	73699999	1722	
	5	73700000	73799999	1489	
	5	73800000	73899999	1128	

Table S7. Chimeric read statistics across Chr3, Chr5, and target regions

	# pairs with R1 on Chr3 and R2 on Chr5	# pairs with R1 on Chr5 and R2 on Chr3
Within whole chromosomes	53965	53916
On targeted 1M sub region<br>Chr3: 148M-149M<br>Chr5: 73M-74M	3	3
On targeted 200K sub region<br>Chr3: 148400K-148600K<br>Chr5: 73500K-73700K	2	3
On targeted sub region<br>Chr3: 148,556,760-148,571,990<br>Chr5: 73,590,780-73,605,850	2	2

SNP phasing

Since we had the deep sequencing data of pool 1, 3, 4, 6 for T35 (Figure 3) and we also had the whole genome sequencing data from T35, we tried to find SNPs from SCS data and genome data, and to phase the SNPs found in WGS. The SCS deep sequencing data had 17,681,828,816 bp, which covered 28 chromosomes with total length of 4,011,842,579bp. The equivalent sequencing depth was 8.8x (we divided the total covered chromosomes' length by 2 when calculated the depth as we calculated whole genome sequencing coverage by dividing by haploid length ~3Gbp rather than diploid length ~6Gbp). Among the covered chromosomes, 8 could be phased, Table S7. The WGS data had 33,377,290,500bp, and the equivalent sequencing depth was 11x. The reads were gone through the first part of the SCS analysis pipeline. Then with the mapped reads whose mapping quality were above 30, we used GATK best practice to find germline SNPs. For WGS data, we filtered the SNPs by keeping only those with depth larger than 10 and quality (QUAL) higher than 30. We further filtered the SNPs in WGS by keeping only those records that were found as COMMON SNP (>= 1% minor allele frequency in at least one 1000Genomes population and was found in two or more people in that population) in dbSNP build149. The records after filtering served as the heterozygous SNPs that were needed to be phased by SCS deep sequencing.

We focused on the homozygous SNPs found in SCS deep sequencing and filtered them by the WGS heterozygous SNPs' coordinates. If one SNP was found in WGS as heterozygous and was also found in one of

the haploid of that chromosome as homozygous, we defined this SNP as concordant SNP. However, if one SNP was defined as concordant SNP in both haploids, which caused conflict in phasing and should be happened in very rare cases, we then defined it as conflict SNP. The number of phased SNPs is calculated by summing up the concordant SNPs in both haploids and subtracting the conflict SNPs. The phased ratio was calculated as the number of phased SNPs dividing by total heterozygous SNPs in WGS. The accuracy was calculated as the number of phased SNPs dividing by total heterozygous SNPs' positions covered by both haploids. The results were shown in Table S9. In total, among 8 homologous chromosomes, 14,398 SNPs were phased. Using the heterozygous SNPs found in WGS to perform phasing, the deep sequencing from SCS phased 5.48% of them and achieved the average accuracy of 97.72%. The errors could be caused by the PCR and sequencing errors from WGS or SCS, but also could be from the uniqueness of this single cell comparing to the bulk cells used in WGS. The low phased ratio was due to the low coverage on the haploids. We checked the IGV results and the bam files of the deep sequencing of SCS and found although the equivalent sequencing depth achieved to 8.8x, the coverage of was low, about 1% for the present chromosomes. The high amount of raw sequencing data was coming from sequencing the covered region for many times. This was due to the extremely low initial amount of DNA and the intrinsic nature of WGA amplification method we used.

Table S8. The chromosomes covered in SCS deep sequencing for sample T35

Chr		1	2	3	4	5	6	7	8	9	10	11	12	13	14	15	16	17	18	19	20	21	22	X	Y
Pool	1																								
	3																								
	4																								
	6																								
can be phased		√	√	√		√	√								√	√	√								
Total chromosomes covered: 28, Total length: 4,011,842,579 bp																									

Wet Lab Protocol

1. Collect 2ml of blood sample using EDTA tubes.
2. Cell culture.
   a) Get two bottles of cell culture medium, use 2ml-syringe to add 25 drops of blood to each bottle of culture medium under sterile environment. Mix it gently and incubate for 69 hours under 37Celsius, 5% CO_2 .
   b) Treated with Colchicine: after 69 hours incubation, use 1ml-syringe to add Colchicin into the cell culture medium until the final concentration reaches 0.2ug/ml. Let it stand for 3 hours.
3. Collect metaphase lymph cells
   a) Get cells: Merge two bottles of culture. Centrifuge at 500g for 5min. Discard supernatant, add 10ml 75mM KCl solution, resuspend the cells gently. Let it stand at RT for 15min.
   b) Add 200ul acetic acid (final concentration: 2%), mix it gently and place it on ice for 30min.
   c) Centifuge at 800g for 5min. Discard supernatant.
   d) Add absolute ethanol:acetic acid (3:1) mixture 10ml, resuspend cells gently. Centrifugate at 800g for 5min. Finish this step as quickly as possible.
   e) Use 2ml 75mM KCl solution to wash the cells. Centrifuge at 800g for 5min to remove ethanol.
   f) Resuspend cells by mixture as the following table. Divide the cells according to the amount of them. Put them in 4Celsius refrigerator overnight.

**Cell resuspension solution recipe**	
Reagents	Volume
1mM EDTA pH8.0	10ul
75mM KCl	9.87ml
1% Triton X100	100ul
100mg/ml Rnase	20ul

   g) Prepare the cell lysis before as the following table.

**Cell lysis recipe**	
Reagents	Volume
75mM KCl	9.7ml
1% Triton X100	100ul
2% Acetic acid	200ul
0.03% Pepsin *	0.003g
* Pepsin is sensitive to pH, it will be inactivated when pH > 4.5. So add pepsin last.	

4. Get single cell and separate chromosomes
   a) Inhale one metaphase cell by microinjection. Put it into the cell lysis prepared before.
   b) Observe the lysis of the cell, after the segregation of chromosomes, suck in the chromosomes and

exhale them to the low-binding PCR tube that contains 20 ul ddH_2O pre-added.

Centrifuge at 13000rpm for 5min. Resuspend the chromosomes by a low-binding tip. And then distribute them into 8 low-binding PCR tubes as the following figure. Pay attention to the minutiae: use the same tip for the whole process, even distribution, before each distribution mix the mixture thoroughly and gently. Evenly distribute the suspension in the tube into "n" partitions (n=8, 16, 24, 32) using ultra-low adsorption pipette tips. Make sure not to discard any tube or pipette tips. Dilution procedure is shown in **Figure S3**.

c) Make two control samples: one is cell lysis solution, the other is ddH_2O used in the distribution process.

5. Whole genome amplification

a) Make sure every tube contains 4.5 ul TE/ddH_2O.

b) Prepare the reaction mix as the following table and mix it thoroughly.

**Fragmentation mix recipe**	
Proteinase K	1 ul
10X single cell lysis & Fragment buffer	16 ul

c) Add the reaction mix into each tube (0.5ul /tube).

d) Incubate DNA mix at 50Celsius for 1 hour. Heat it to 99Celsius for 4min precisely. Place tubes on ice immediately and then centrifuge.

e) Add 1ul 1x single cell library preparation buffer to each tube.

f) Add 0.5 ul Library Stabilization Solution to each tube.

g) Mix them thoroughly, and place them at 95Celsius for 2min.

h) Place them on ice to cool down them and then centrifuge.

i) Add 0.5 ul Library Preparation Enzyme to each tube, mix them thoroughly and then centrifuge.

j) Incubate them as the following table.

**Incubation parameters**	
Temperature (Celsius)	Time
16	20min
24	20min
37	20min
75	5min
4	Hold

k) Centrifugate them thoroughly. The products of this step can be amplified immediately or store at -20Celsius for 3 days.

l) Amplification preparation: Add the reagents to the 8 tubes made above (each tube should contain

7ul sample). The final volume for each tube should be 37.5ul.

WGA4 PCR amplication Reagents	
Regents	Volume
10x Amplication Master Mix	3.75ul
Water (molecular level)	24.25ul
WGA DNA polymerase	2.5ul

m) Mix them thoroughly, centrifuge and then run PCR as the following table.

WGA4 PCR amplication program		
Temperature (Celsius)	Time	
95	3min	
94	30sec	25 cycles
65	5min	
4	Hold	

n) Collect the products. Fragments should be about 300bp. Purify by column purification/Ampure xp beads.

6. WGA3 amplification

a) Add 10 ul WGA4 products to 8 tubes respectively.

b) Add the PCR reaction mix as the following table. The final volume for each tube should be 75 ul.

WGA3 PCR amplication Reagents	
Regents	Volume
Nuclease-Free water	49.5ul
10x Amplication Master Mix	7.5ul
10x dNTP Mix	3.0ul
WGA DNA polymerase	5.0ul

c) Mix them thoroughly, centrifuge and then run PCR reaction as the following table.

WGA3 PCR amplication program		
Temperature (Celsius)	Time	
95	3min	
94	15sec	14 cycles (20cycles)
65	5min	

d) Purify the products by column purification.

7. Build library

a) DNA end repair.

i. Prepare reaction mix as following table.

DNA end repair Reagents

Regents	Volume
10x PNK buffer	5 ul
dNTP (2mM)	10 ul
T4 DNA polymerase	2.5 ul
T4 PNK	2.5 ul
Klenow Fragment	0.5 ul
DNA	29.5 ul
Total	50 ul

ii. Incubate tubes as the program: 20Celsius for 30min.

iii. Use 1.8:1 ratio of Ampure Beads to purify products. Get 34 ul-elution.

b) Add Adenine

i. Prepare reaction mix as following table.

**Adding Adenine Reagents**	
Regents	Volume
10x NEB2 buffer	5 ul
dATP (1mM)	10 ul
Klenow exo	3 ul
DNA	32 ul
Total	50 ul

ii. Incubate tubes as the program: 37Celsius for 30min.

iii. Use 1.8:1 ratio of Ampure Beads to purify products. Get 29 ul-elution.

c) Adapter ligation

i. Prepare reaction mix as following table.

**Adapter Ligation Reagents**	
Regents	Volume
2x quick ligase buffer	50 ul
Adapter (diluted)	20 ul
Quick ligase	3 ul
DNA	27 ul
Total	100 ul

ii. Incubate tubes as the program: 20Celsius for 15min. Add 3 ul LISER then incubate at 37Celsius for 15min.

iii. Use 1.8 Ampure Beads to purify products. Get 25 ul elution.

d) Sequencing index ligation and PCR amplification

i. Prepare reaction mix as following table.

**Index Ligation and PCR Reagents**	
Regents	Volume
Phusion master mix	25 ul
DNA	23 ul
Pi	1 ul
Index	1 ul
Total	50 ul

ii. Mix them thoroughly, centrifuge and then run PCR as the following table.

**Index ligation and PCR amplication program**		
Temperature (Celsius)	Time	
98	1min	
98	20sec	25 cycles
65	30sec	
72	30sec	
72	5min	
4	Hold	

References

1. Bolger, A.M., Lohse, M. and Usadel, B. (2014) Trimmomatic: a flexible trimmer for Illumina sequence data. *Bioinformatics*, btu170.
2. Schmieder, R. and Edwards, R. (2011) Quality control and preprocessing of metagenomic datasets. *Bioinformatics*, **27**, 863-864.
3. Li, H. and Durbin, R. (2010) Fast and accurate long-read alignment with Burrows–Wheeler transform. *Bioinformatics*, **26**, 589-595.
4. Meyer, L.R., Zweig, A.S., Hinrichs, A.S., Karolchik, D., Kuhn, R.M., Wong, M., Sloan, C.A., Rosenbloom, K.R., Roe, G. and Rhead, B. (2013) The UCSC Genome Browser database: extensions and updates 2013. *Nucleic acids research*, **41**, D64-D69.
5. McKenna, A., Hanna, M., Banks, E., Sivachenko, A., Cibulskis, K., Kernytsky, A., Garimella, K., Altshuler, D., Gabriel, S. and Daly, M. (2010) The Genome Analysis Toolkit: a MapReduce framework for analyzing next-generation DNA sequencing data. *Genome research*, **20**, 1297-1303.
6. Krzywinski, M.I., Schein, J.E., Birol, I., Connors, J., Gascoyne, R., Horsman, D., Jones, S.J. and Marra, M.A. (2009) Circos: An information aesthetic for comparative genomics. *Genome Research*.
